# Training in the use of basic functions of the daVinci Xi^®^ robot: a comparative study of residents’ skills

**DOI:** 10.1007/s13304-025-02150-z

**Published:** 2025-03-15

**Authors:** Gaspare Cannata, Nicola Leone, Antonio Salzano, Fabrizio Rebecchi, Mario Morino

**Affiliations:** 1 Department of Surgical Sciences, University of Turin, Turin, Italy; 2 Umberto Primo Hospital, Nocera Inferiore, Salerno Italy; 3 Department of Surgical Sciences, University of Torino, C.So Dogliotti 14, 10126 Turin, Italy

**Keywords:** Robotic surgery, daVinci Xi robot, Minimal invasive surgery, Robotic training

## Abstract

The rapid spread of the robotic surgical system has not been accompanied by an equally rapid creation of standardized training courses for the use of this technology.

The purpose of our study was to evaluate skill acquisition in the handling and use of the daVinci Xi by comparing two groups of surgical residents. Surgical residents from the University of Turin were enrolled. The participants were divided into two groups: Group A: residents who had participated in at least 8 robotic surgical procedures, and Group B: residents who had never attended robotic surgery. All were administered two instructional videos on the patient cart and console exercises to be performed. Subsequently, the residents were tested and recorded to be evaluated by a senior surgeon experienced in robotic surgery, according to a previously assessed evaluation score. The time of the procedure was also recorded for each test. Patient cart exercises were completed by all participants. We found statistically significant differences between two groups for the first (*p* = 0.0000) and third (*p* = 0.0002) patient cart tests and for every test on the surgeon's console except the endoscope handling exercise. Group A scored higher on the patient cart exercises, and the difference reached statistical significance (*p* = 0.0001). The placement of a single hand-sewn knot on the silicone suture pad was the only exercise that was not fully completed by all participants and showed no statistical difference. The correlation analysis between surgical experience and final score was significant in Group A. The daVinci Xi robotic platform can be properly operated in its basic functions by young surgeons after a short training program even in the absence of previous exposure to robotic clinical procedure.

## Introduction

Over the past fourteen years, robotic surgeries have seen a dramatic increase. More than 1.75 million robotic surgeries have been performed [1]. Currently, the da Vinci System^®^ (Intuitive Surgical Inc.) is the most widely used system for performing robot-assisted laparoscopic surgery [2, 3]. This increased adoption has highlighted the need for an effective training program in this field, to ensure that operators possess adequate surgical skills and for patient safety [4, 5]. However, there is still no universally shared robotic training program in Europe, also because in recent years different surgical robotic platforms have emerged.

Beyond the surgeon and assistant, all operating room personnel (nurses, technicians, etc.) must be properly trained on this specific equipment. Currently, no standardized training criteria are established for surgeons, nurses or operating room technicians regarding the appropriate operation of robotic surgical systems in the operating room [6]. Several surgical societies are trying to provide recommendations for training in robotic surgery, however, calling for standardization and consensus on the type of training and curriculum needed to independently initiate robotic surgery on patients [7–9]. Indeed, the most prevalent robotics training programs are currently offered by the manufacturers of specific robotic platforms. For instance, Intuitive, a leading robotics company, provides and mandates comprehensive training for its surgical robot systems.

Our study aims to compare the skill acquisition of two resident groups in managing and using the Da Vinci Xi, assessing their performance based on prior experience and year of residency.

## Materials and methods

Thirty surgical residents from the University of Turin, were divided into two groups: Group A, residents who had participated in at least 8 robotic surgical procedures, and Group B, residents who had never attended robotic surgery.

All participants completed basic daVinci Xi^®^ robot tasks, divided into docking and console phases. These phases involved a training session followed by a practice session.

The training session comprised two video tutorials. The first one was on components overview: a certified expert in robotic technologies explained the platform's components (patient-side cart and console) and their use (Table 1). The second video was focused on exercise demonstration: participants watched demonstration videos on the basic exercises they would perform (Table 2). During each practice session, only the individual trainee was admitted to the operating room. All other trainees were excluded. The young surgeon was required to perform a series of practical exercises, both at the cart and console, as detailed in Table 2. Each individual practice session was videorecorded for subsequent evaluation, and the time taken to complete each exercise was recorded. All participants completed and signed an informed consent form about the testing procedures before participation.

**Table 1:** Training session (part 1): consisted of watching two video tutorials regarding the platform components (including the patient-side cart and console) and its use

Patient-side cart	1. Components and functionality of the arms: -Use of the instrument clutch to perform fine movements around the remote center and to insert the tools; -Use of the port clutch to perform wider movements of the arm, hook the trocar to the arm and/or move it away, or “make a tent” to give more maneuverability to the instruments in operation; -“grab and move” functionality; -Use of the “patient clearance” and “instrument reach” buttons, which control the outermost elbow of the arm to move it away or bring it closer to the patient 2. Components and functionality of the endoscope (0° or 30°): eye change button and aiming; button to take snapshot; light button 3. Approach of the robot cart after correct positioning of the patient on the operating bed and method of anchoring the arms to the trocars 4. Insertion of the endoscope and aiming at the reference anatomy with consequent arrangement of the arms to facilitate their anchoring to the trocars 5. Insertion and removal of the instruments in view, also explaining the meaning of memory on reintegration
Console	1. buttons located on the left in the armrest, useful for positioning the console itself to be as ergonomic as possible thanks to the possibility of raising or lowering the steric viewer, modifying the inclination and height of the armrest, bringing the pedal board 2. pedalboard: -Optics pedal and its operation by using the masters; -Clutch pedal that allows the masters to be mobilized and repositioned, dissociating them from the movement of the instruments when they are too close to each other or in uncomfortable positions; -Switch pedal to exchange the instrument in control; -Electrified instrumentation control pedals (mono–/–bi-polar) 3. the masters and the steric viewer and how to take control of the instruments through them

**Table 2:** Training session (part 2): consisted of watching a video tutorial regarding the exercises (three at the patient-side cart and five at the console), which participants performed in the practical session

Patient-side cart	1. Attachment of the trocar to the endoscope arm, its insertion and aiming at the reference objective 2. Attachment of the other arms to the trocars and insertion of the three remaining instruments in view 3. Replace all instruments and alternate the eye of the endoscope
Console	1. Repositioning of the optics keeping the instruments in view: the participant was given eight voice commands on moving the endoscope (right and left, up and down, approach and departure, rotation to the right and rotation to the left) 2. Using two operating tools to always keep in view, take a gauze inside the operating field, reachable only by using the clutch, and then to place it inside a basket, without moving the optics 3. Handling of instruments within the operating field (take four gauzes and place them in a basket, always keeping the 2 instruments in view) 4. Use of the switch pedal and therefore of the third arm, which was used to keep a gauze suspended, thus allowing, through the use of the other 2 tools, to pick up a smaller one and place it in a basket 5. Affixing a simple 2/0 silk stitch, making 3 knots, on a horizontal defect in a silicone intestine model, suitably fixed to the bottom of the pelvic box. The participant was asked to perform the exercise in no more than 7 '

A robotic-experienced surgeon, blinded to the residents’ group affiliation, evaluated video recordings of the practical exercises performed by the participants.

Each exercise evaluation considered both quantitative (execution time) and qualitative aspects to assess the learning process and skill acquisition. The qualitative aspects are reported in Table 3 and in Table 4.

**Table 3:** Scores assigned for patient-side cart exercises

Exercise/skill	Scores
1. Use of the arms and endoscope targeting	
Use of instrument clutch	0 to 2: 0 not used; 1 if used only in one phase of the exercise (e.g., introduction of the endoscope); 2 own use during all phases of arm positioning
Use of port clutch	0 to 2: 0 not used; 1 if used only in one phase of the exercise (e.g., trocar attachment); 2 proper use during all positioning phases arm
Attachment of the trocar	0 to 1: 0 trocar engagement not performed (at least 3 unsuccessful attempts to engage the trocar); 1 done correctly
Endoscope targeting	0 to 1: 0 aiming not performed (or failed after 3 attempts); 1 pointing correctly performed
2. Insertion of tools	
Attachment of the trocar	0 to 1: 0 trocar engagement not performed (at least 2 unsuccessful attempts to engage the trocar); 1 done correctly
Correct insertion and positioning of instruments	0 to 2: 0 if instruments introduced out of the visual field; 1 if introduction performed correctly, but instruments not triangulated or not fully visible; 2 if introduction was done correctly with triangulated instruments and in vision
3. Tool replacement and gearbox eye endoscope	
Correct re-insertion of instruments after replacement	0 to 2: 0 repositioning of the instruments out of the visual field; 1 reintroduction of instruments not using the memory function; 2 correct reintroductions using the memory function
Use of eye change control	0 to 1: 0 key change eye not identified; 1 key change eye identified

**Table 4:** Scores assigned for console exercises

Exercise/skill	Scores
1. Endoscope use	0 to 8: one point for each command performed correctly
2. Clutch pedal use	0 to 2: 0 clutch function not used/operation not completed; 1 correct use of the clutch with errors in the coordination of the instruments (e.g., loss of grip, collision with the basketball); 2 correct use and good clutch coordination of tools
3. Handling of instruments within the operating field	0 to 2: 0 exercise not completed in 3’ or loss of both instruments from the visual field; 1 exercise performed not always keeping two instruments in view; 2 if performed while keeping always in vision the instruments Only one penalty point was awarded for loss of grip or collision against the basketball
4. Use of switch pedal and third arm	0 to 2: 0 if loss of 2 instruments from the visual field; 1 if loss of a single instrument from the visual field; 2 exercise performed correctly with all instruments always in view Only one penalty point has been assigned if errors are made in the coordination of instruments (e.g., loss catch, bump against basketball)
5. Simple stitch affixing and knotting -Stitch affixing -Knotting	0 to 2: 0 exercise performed beyond the limit of 7’ (however it was considered as the exercise execution time) or failure in passing needle; 1 passing needle without coordination of second instrument or not correctly addressing the margins of the silicone model; 2 needle passage performed with fluid movement using both tools and correctly addressing the margins of the silicone model Only 1 penalty point was awarded if the following occurred during the test: breakage of the needle or thread, loss of silicone model 0 to 3: one point for each knot performed within the established time limit

The primary outcome of the study was to assess the participants’ ability to perform basic procedures on the da Vinci Xi^®^ robot platform (having never used it before) within a predetermined amount of time (based on the evaluating surgeon's execution time, experienced in robotic surgery) while also assessing the scores achieved by each of them. This study evaluated the use of the arms and endoscope targeting, the insertion of tools and tool replacement and gearbox eye endoscope for the patient-side cart exercises. For console exercises, it evaluated endoscope use, clutch pedal use, handling of instruments within the operating field, use of switch pedal and third arm and simple stitch afflicting and knotting. Scores were assigned based on criteria outlined in Table 3 and Table 4. Secondary outcomes included the following: execution times of completed procedures (based on the evaluating surgeon's execution time, experienced in robotic surgery), blinded qualitative assessment of acquired skills (based on predetermined scoring criteria), and impact of residency training years and previous robotic experience on both execution times and overall evaluation.

The statistical analysis was conducted with STATA 14.1 software. Shapiro–Wilk test was performed to check for distributive normality. Independent sample t-tests were performed on all exercise times. The Kruskal Wallis test was performed for a non-parametric analysis of variance based on ranks. A Spearman test was calculated to verify the correlation between the quantitative variable “years of training” and the variables “total score at the patient cart,” “total score at the console” and “time” of all tests. For each exercise, a linear regression was calculated with the dependent variable “years of training” and the independent variable “time,” in which we found the significance of Spearman’s test. We also ran a regression between the total score of exercises at the console and years of training and between the total score of exercises at the cart and years of training.

## Results

From the Department of Surgical Sciences at the University of Turin, 30 surgical trainees were recruited. 15 were assigned to Group A, and 15 to Group B. 28 trainees were in general surgery, 1 in otolaryngology, and 1 in maxillofacial surgery. Residents in Group A were distributed across training years as follows: 4 (26.7%) in the sixth year, 7 (46.7%) in the fifth, 3 (20.0%) in the fourth, and 1 (6.7%) in the third year. Group B residents were distributed as follows: 5 (33.3%) in the fifth year, 2 (13.3%) in the fourth, 1 (6.7%) in the third, 3 (20.0%) in the second and 4 (26.7%) in the first year. The general surgery residency course lasted six years.

**Fig. 1:**
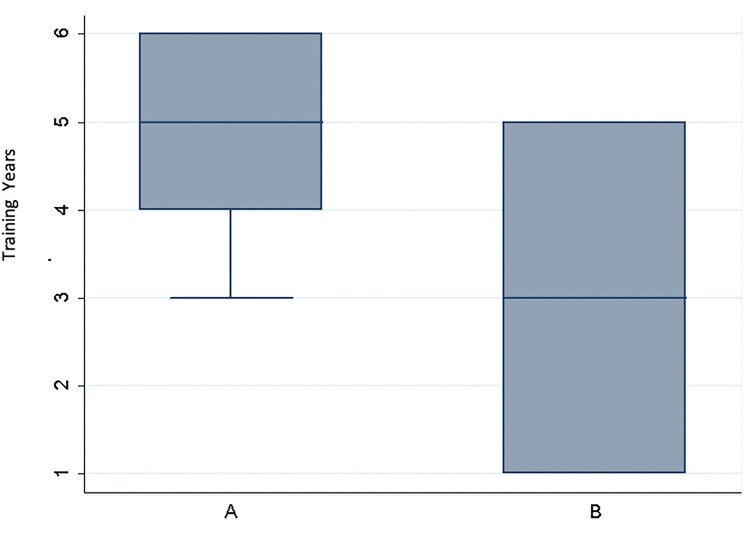
Boxplot years of training Group A and Group B

Residents in Group A had significantly more years of training compared to Group B (*p* = 0.0008). The median years of training were 5 (SD 1) for Group A and 3 (SD 1.7) for Group B (Fig. 1).

All study participants concluded the patient cart and console exercises.

**Fig. 2:**
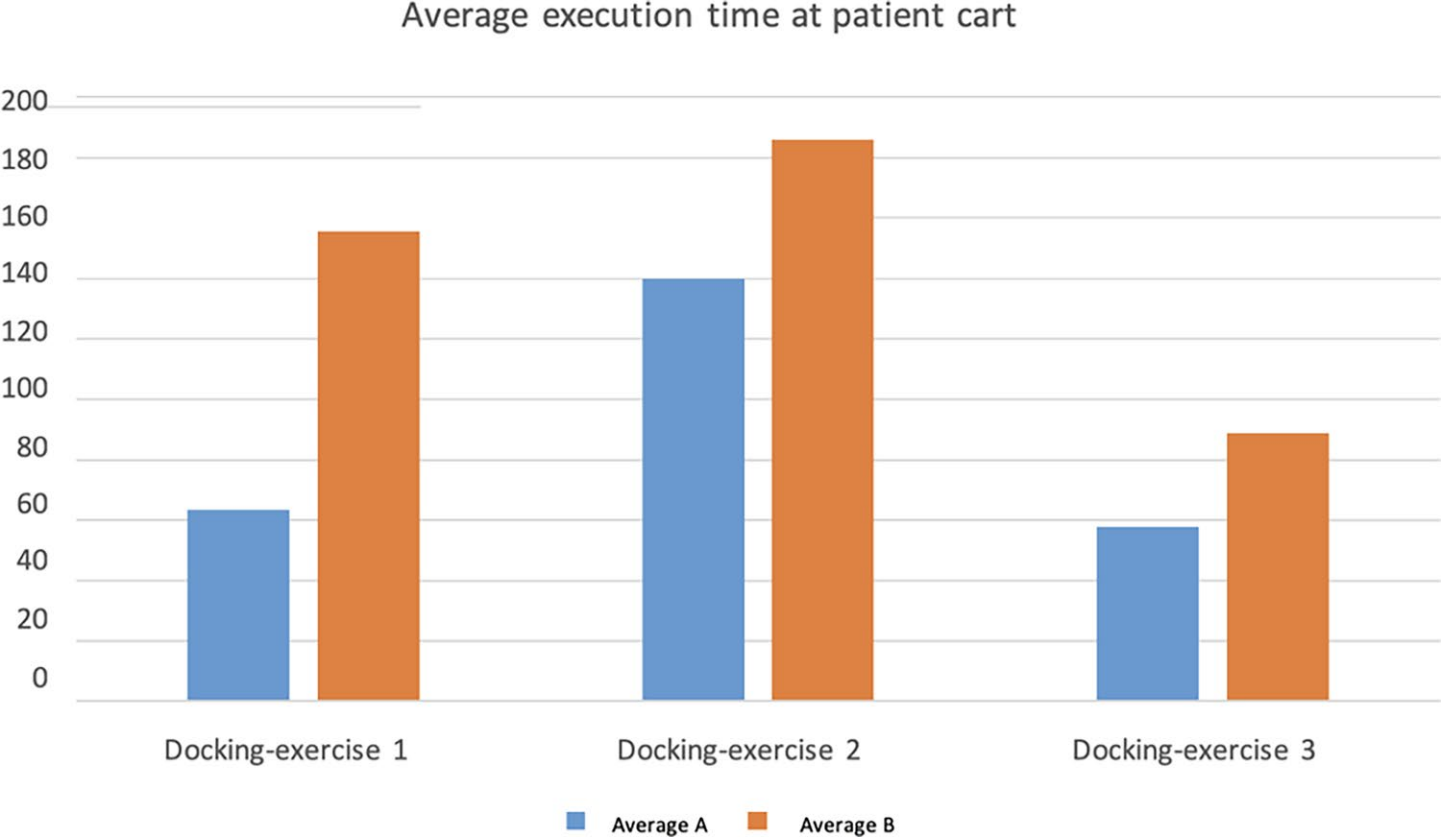
Averages of patient cart exercise times

The execution times of the first (*p* = 0.0000) and third (*p* = 0.0002) exercises at patient cart differed significantly between the groups. For the first exercise (use of arms and endoscope targeting), Group A completed it in an average of 63.5 s (SD 35.4), while Group B took an average of 155.5 s (SD 61) (Fig. 2). Similarly, the third exercise (instrument replacement and endoscope eye change) showed a significant difference, with Group A averaging 57.9 s (SD 20.7) and Group B averaging 88.8 s (SD 18.8). Notably, no significant difference was observed in the execution times of the second exercise (instrument insertion) (*p* = 0.0934), suggesting minimal impact on the second step from the instrument removal in the third exercise (Table 5).

**Table 5:** data analysis, times and scores on the patient's cart

	Group A			Group B			*P*-value
	Median	Mean	SD	Median	Mean	SD	
1. Use of the arms and endoscope targeting:							
Time(s)	62.0	63.5	35.4	144.0	155.5	61.0	0.0000
Use *port clutch*	2	2	0	2	1.7	0.6	0.0730
Use *instrument clutch*	2	1.9	0.3	2	1.4	0.7	0.0136
Attachment of the trocar	–	–	–	–	–	–	0.0347
Aiming	–	–	–	–	–	–	0.0061
2. Insertion of tools:							
Time(s)	153.0	139.8	45.5	162.0	186.0	92.4	0.0934
Attachment of the trocar	–	–	–	–	–	–	0.3173
Correct insertion and positioning of instruments	2	1.9	0.4	2	1.5	0.5	0.0502
3. Tool replacement and gearbox eye endoscope							
Time(s)	51.0	57.9	20.7	89.0	88.8	18.88	0.0002
Correct re-insertion of instruments after replacement	2	1.7	0.5	1	1.3	0.5	0.0309
Use of eye change control	–	–	–	–	–	–	–
Total score	11	11.40	0.63	9	9.13	1.46	0.0001

Concerning the execution time of manual tasks, statistically significant differences were observed for exercises two (use of clutch pedal) (*p* = 0.0095), three (manipulation of instruments within the operating field) (*p* = 0.0072) and four (use of switch pedal and third arm) (*p* = 0.0065) (Table 6).

**Table 6:** data analysis, times and scores on the console

	Group A			Group B			*P*-value
	Median	Mean	SD	Median	Mean	SD	
1. Endoscope use							
Time(s)	56	51.9	9.4	45	45.7	12.3	0.1281
	7	6.4	1.6	7	6.7	1.5	0.5309
2. Clutch pedal use:							
Time(s)	28.0	34.3	18.7	60	80.4	61.2	0.0095
Management joystick via clutch	2	1.9	0.4	2	1.7	0.5	0.3694
3. Handling of instruments within the operating field:							
Time(s)	79	79.20	21.00	100	113.70	41.71	0.0072
Movement skills	2	1.6	0.5	2	1.5	0.7	0.7735
4. Use of switch pedal and third arm:							
Time(s)	80	85.60	40.40	117.00	142.30	63.00	0.0065
Arms manage-ment & switch	2.00	1.70	0.50	1.00	1.40	0.60	0.1217
5. Simple stitch affixing and knotting:							
Time(s)	182.00	208.70	110.10	281.00	299.00	109.40	0.0323
Stitch affixing	2.00	1.40	0.70	1.00	0.90	0.80	0.0771
Knotting	3.00	2.30	1.10	2.00	2.00	1.10	0.3330
Total score	15.00	15.33	3.09	15.00	14.40	2.58	0.3801

**Fig. 3:**
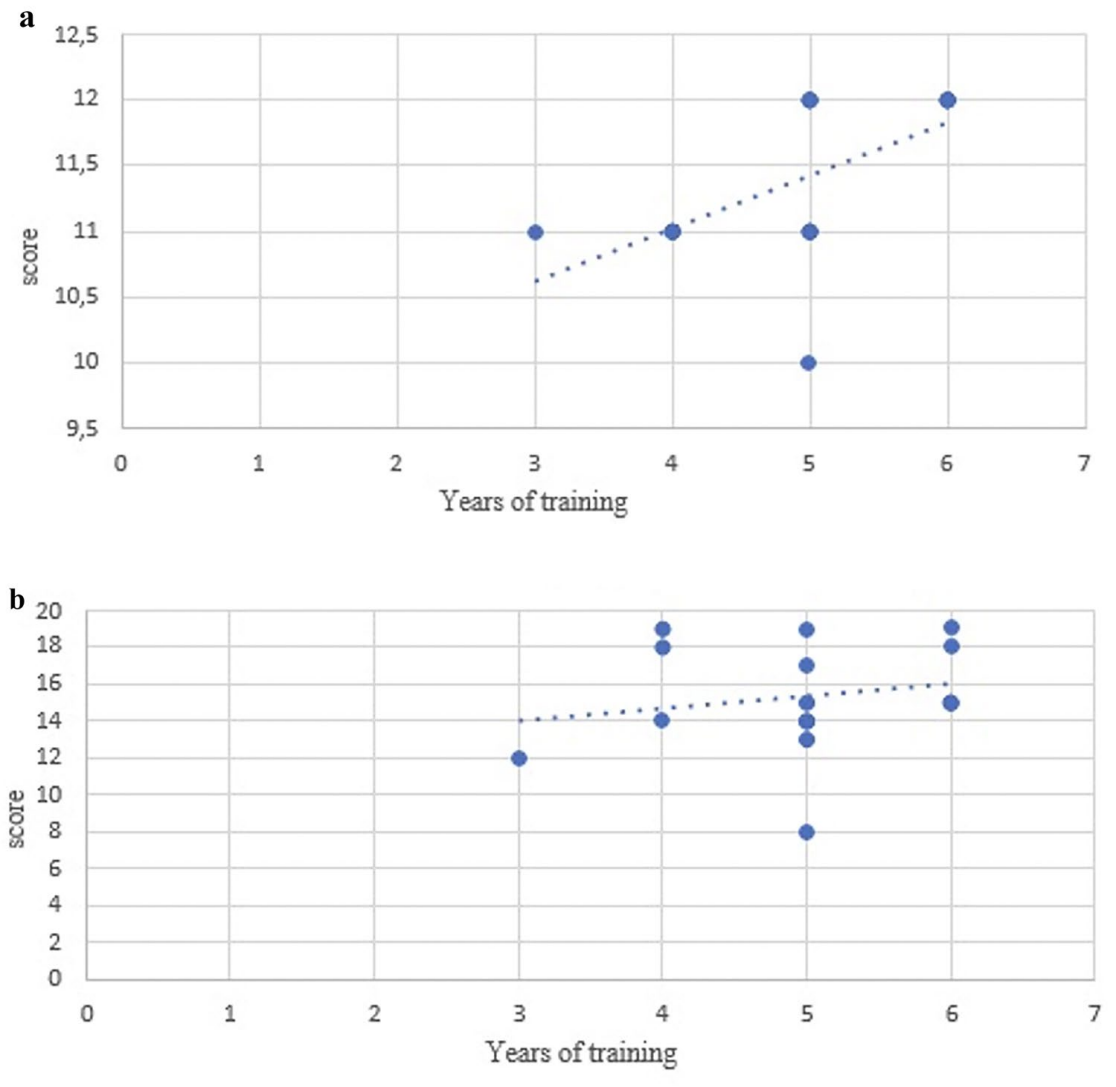
**a** Correlation of total patient cart scores/years of training Group A. **b** Correlation of total console scores/years of training Group A

**Fig. 4:**
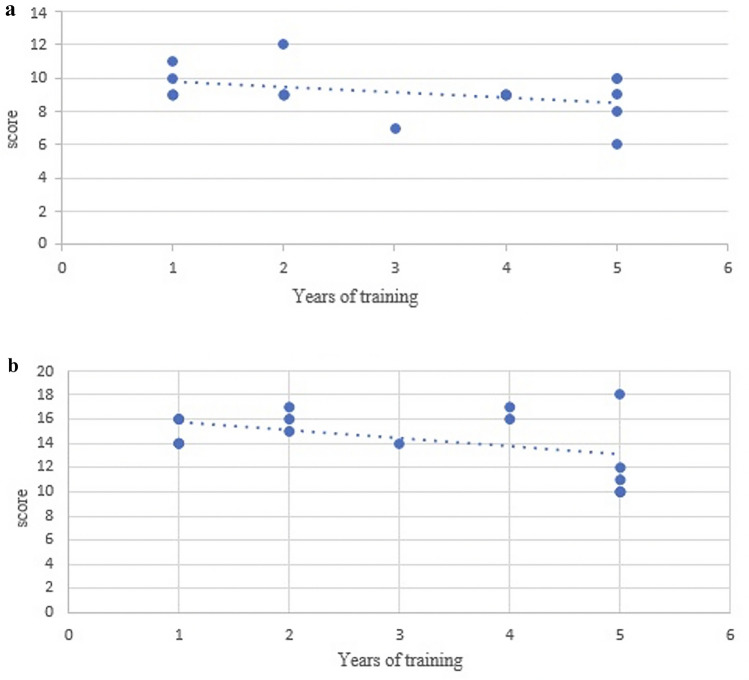
**a** Correlation of total patient cart scores/years of training Group. **b** Correlation of total console scole/years of training Group B

A Spearman's rank correlation coefficient analysis revealed a significant positive correlation between “years of training” and “patient cart exercise score” (Fig. 3a and Fig. 4a) for Group A (*p* = 0.0083), but not for Group B (*p* = 0.2448). Conversely, no significant correlations were observed between “years of training” and “console exercise score” (Fig. 3b and Fig. 4b) in either group. A significant positive correlation was found between “years of training” and “time” for exercises 1 (*p* = 0.0051) and 3 (*p* = 0.0053) on patient cart for group A. Similarly, a positive correlation was observed between “years of training” and “time” for exercise 4 at console (*p* = 0.0382) (Table 7).

**Table 7:** Analysis of the correlation between total scores-single times of the exercises and years of training

	Group A		Group B	
	RHO	*p* value	RHO	*p* value
Years of training and console scoring	1.520139	0.4332	0.3162	0.2508
Years of training and patient-cart scoring	0.6534	0.0083	0.3201	0.2448
Years of training and exercise 1 patient-cart time	− 0.6824	0.0051	0.3677	0.1776
Years of training and exercise 2 patient-cart time	− 0.0987	0.7265	0.0645	0.8192
Years of training and exercise 3 patient-cart time	− 0.6793	0.0053	0.0434	0.878
Years of training and exercise 1 console time	− 0.2889	0.2963	0.1672	0.5514
Years of training and exercise 2 console time	0.2283	0.4131	0.1863	0.5062
Years of training and exercise console time	− 0.3719	0.1723	0.1199	0.6704
Years of training and exercise 4 console time	− 0.5389	0.0382	0.2732	0.5382
Years of training and exercise 5 console time	− 0.3361	0.2206	0.3476	0.2042

Linear regression analysis revealed a significant association between dependent variable “years of training” and the “time” taken to complete the first (*p* = 0.0017) and third (*p* = 0.0029) patient card exercises for Group A. The regression analysis was also significant for group A for the fourth exercise at the console (*p* = 0.0145) (Table 8).

**Table 8:** Regression analysis of statistically significant parameters to Spearman’s test

Group A			Group B	
	*p* value	Years of training coefficient	*p* value	Years of training coefficient
Years of training and patient-cart scoring	0.0283	0.36	0.1667	–
Years of training and exercise 1 patient-cart time	0.0017	− 0.26	0.3227	–
Years of training and exercise 3 patient-cart time	0.0029	− 0.15	0.9361	–
Years of training and exercise 4 patient-console time	0.0145	− 0.25	0.5298	–

## Discussion

The advent of robotic technology in minimally invasive surgery has been accompanied by a significant rise in publications, with over 4000 studies showing its potential advantages over laparoscopy. These potential benefits include improved stereoscopic vision, preservation of natural hand–eye coordination, and enhanced dexterity due to elimination of the fulcrum effect caused by abdominal wall [10]. Despite these potential advantages, the superiority of robot- assisted surgery over laparoscopy in terms of intraoperative, perioperative and oncological outcomes remains unproven [11].

A most important issue in recent years has been the need for effective, robotic surgery-specific training programs to ensure adequate surgical skills and patient safety [4, 5]. This is particularly important for residents. Training young surgeons in the use of robotic platforms, both at the patient cart and the console, is essential to optimize system utilization and enhance patient safety. Various devices have been developed to assess technical skills in both open and laparoscopic surgery subsequently validated by scientific evidence [12, 13]. The GOALS–score (GlObal Assessment tool for evaluation of intraoperative Laparoscopic Skills) has been developed and validated for intraoperative evaluation of laparoscopic skills in different surgical procedures [12, 14]. The need to develop similar assessment device, also for robotic surgery, is mandatory in order to create standardized training courses [15, 16]. The GEARS-score (Global Evaluative Assessment of Robotic Skills) [17] aims to provide a valid, reliable, and reproducible assessment of intraoperative robotic surgical skills. Proficiency-Based Progression (PBP) training methodology is an objective and validated training methodology that demonstrated to increase the clinical and surgical outcomes of 40 to 60% compared to the traditional way of training [18].

In order to evaluate the feasibility and reproducibility of the basic functions of the robotic platform, currently used in clinical practice (daVinci Xi^®^–Intuitive Surgical, Sunnyvale, CA, USA—Ab Medica SPA, Milan, Italy), by surgical residents during a surgical residency program, we compared two groups of young surgeons with varying levels of training and experience in robotic technology.

Our study shows that all participants, after a training video tutorial, regardless of their group, were able to perform the proposed surgery at the patient-cart of the daVinci Xi^®^.

Our evaluation appears to have effectively distinguished the more experienced group in robotic surgery from the inexperienced group. The difference emerged for the tasks analyzed in the patient-cart both in the evaluation of individual exercises and in terms of total score. In fact, Group A scored significantly better in total score in the use of the patient-cart (*p* = 0.0283) and a better rating in the use of some components of the arms (Table 8).

Multivariate analysis revealed a significant correlation between years of surgical training and the total score obtained in the patient–cart exercises for Group A. Specifically, the regression analysis indicated that each additional year of training experience increased the score by 0.35 points (coefficient of years of experience = 0.35). These results are in line with Goh's previous work [17] who reported similar results regarding the GEARS score’s ability to differentiate young surgeons based on their training years in real-world surgical setting.

In Group B 46.7% of surgeons residents completed the aiming task only after the third attempt. In the first three attempts, although they correctly identified the aiming button, they did not hold it down until completion. This suggests potential limitations in the user feedback mechanisms related to the aiming function from robot, despite the button being readily identifiable and the task instructions clear. Regression analysis revealed an inverse correlation between years of training and execution times for the first and third patient cart exercises in Group B. This indicates that additional years of training experience were associated with faster completion times for these exercises.

The difference in scores at patient cart exercises between Groups A and B likely relates to the varying distribution of residents by year of specialization across the groups. However, even minimal prior robotic experience appears to exert a further influence on performance.

In contrast to the patient cart tasks, those at the console showed greater uniformity between the two groups in terms of the expert's qualitative assessment of both individual exercise and overall performance. This suggests that the basic console functions were intuitive for all participants, even though not everyone could complete the final test within the allotted time.

Not all participants in Groups A and B completed the console exercise designed to assess purely surgical skills (suture placement and knot tying with three knots) within the allocated time. In Group A, this may be attributed to varying levels of experience with the robotic platform and years of training, as evidenced by two young surgeons (one in third year and one in fifth year) failing to complete the knot tying task. Similarly, in Group B, differing levels of surgical experience likely contributed to 9 participants (60%) not completing the knot tying on their first attempt. The most common difficulty reported by both groups for this exercise was thread or needle breakage due to excessive pulling, which aligns with the previously identified limitation of robotic surgery: the lack of tactile and haptic feedback [19]. Our findings support this limitation.

Our study revealed that while participants completed the required tests, the majority encountered difficulties primarily related to operating the patient cart. These challenges highlight the need for targeted and more intensive training, as video tutorials alone proved insufficient. Multivariate analysis revealed that years of experience correlated with faster execution times for specific patient cart exercises, potentially mitigating one of the limitations reported in the literature: increased operative time compared to traditional surgery, especially during the initial learning curve. Regarding the exercises at the console, although a difference in execution time persisted, participants did not demonstrate systematic difficulties in execution.

The console was more intuitive than the patient cart, probably because these basic exercises are more closely related to the surgical act unlike those related to the patient cart and are less related to the specific experience of the robot and independent of the year of specialization except for execution time. Further explanation could be the concept of "gamefication" of the surgical act, a feature discussed in the robotic surgical literature and to which new generations of surgeons are more predisposed. Recently, robotic simulators have been introduced for young surgeons to improve operational skills. These simulators have undergone various validation studies but did not show validity in transferring the skills obtained from the simulator to the operating room [20–22].

The rating scale adopted in this study could be used as a tool for surgical residents to monitor their progress in specific skills. However, further studies are necessary to evaluate how training improvement translate into actual surgical performance.

The small sample size and the heterogeneity of the control group regarding previous experience with robotic surgery are the limitation of our study. To address these limitations, we plan to conduct a follow-up study with the same participants. After a defined period, they will undergo the same practice tests, allowing us to evaluate potential improvements in skill acquisition while maintaining the current training program.

## Conclusion

In conclusion, the use of the daVinci Xi^®^ robotic platform is manageable in its basic functions by young surgeons in training after a short training. This is particularly evident considering that one group of participants was totally inexperienced in this technology.

Training related to the patient-side cart appears to be more affected by the technical characteristics of the current platform, which will likely require further training improvement. The surgical console appears to be more manageable, even with little training, regardless of previous surgical and robot-specific experience, confirming the advances in biomedical engineering in this area.

Overcoming the limitations related to the absence of tactile and haptic feedback and reducing the time of use are closely related to practical training courses regardless of theoretical training.

Further studies are needed to validate and standardize a training model to reduce the learning curve times in clinical practice.

## Data Availability

The data in the study are available for consultation.
